# Highly Pathogenic Avian Influenza Virus H5N1 Infection in a Long-Distance Migrant Shorebird under Migratory and Non-Migratory States

**DOI:** 10.1371/journal.pone.0027814

**Published:** 2011-11-22

**Authors:** Leslie A. Reperant, Marco W. G. van de Bildt, Geert van Amerongen, Debbie M. Buehler, Albert D. M. E. Osterhaus, Susi Jenni-Eiermann, Theunis Piersma, Thijs Kuiken

**Affiliations:** 1 Department of Ecology and Evolutionary Biology, Princeton University, Princeton, New Jersey, United States of America; 2 Department of Virology, Erasmus Medical Centre, Rotterdam, The Netherlands; 3 Animal Ecology Group, Centre for Ecological and Evolutionary Studies, University of Groningen, Groningen, The Netherlands; 4 Swiss Ornithological Institute, Sempach, Switzerland; 5 Royal Netherlands Institute for Sea Research, Texel, The Netherlands; Centers for Disease Control and Prevention, United States of America

## Abstract

Corticosterone regulates physiological changes preparing wild birds for migration. It also modulates the immune system and may lead to increased susceptibility to infection, with implications for the spread of pathogens, including highly pathogenic avian influenza virus (HPAIV) H5N1. The red knot (*Calidris canutus islandica*) displays migratory changes in captivity and was used as a model to assess the effect of high plasma concentration of corticosterone on HPAIV H5N1 infection. We inoculated knots during pre-migration (N = 6), fueling (N = 5), migration (N = 9) and post-migration periods (N = 6). Knots from all groups shed similar viral titers for up to 5 days post-inoculation (dpi), peaking at 1 to 3 dpi. Lesions of acute encephalitis, associated with virus replication in neurons, were seen in 1 to 2 knots per group, leading to neurological disease and death at 5 to 11 dpi. Therefore, the risk of HPAIV H5N1 infection in wild birds and of potential transmission between wild birds and poultry may be similar at different times of the year, irrespective of wild birds' migratory status. However, in knots inoculated during the migration period, viral shedding levels positively correlated with pre-inoculation plasma concentration of corticosterone. Of these, knots that did not become productively infected had lower plasma concentration of corticosterone. Conversely, elevated plasma concentration of corticosterone did not result in an increased probability to develop clinical disease. These results suggest that birds with elevated plasma concentration of corticosterone at the time of migration (ready to migrate) may be more susceptible to acquisition of infection and shed higher viral titers—before the onset of clinical disease—than birds with low concentration of corticosterone (not ready for take-off). Yet, they may not be more prone to the development of clinical disease. Therefore, assuming no effect of sub-clinical infection on the likelihood of migratory take-off, this may favor the spread of HPAIV H5N1 by migratory birds over long distances.

## Introduction

Although wild bird species susceptibility to infection and disease caused by highly pathogenic avian influenza virus H5N1 (HPAIV H5N1) has been studied [1]–[5], their role in dispersing the virus over long distances is difficult to assess. In Western Europe, the route of introduction of the virus was most likely associated with migratory or within-winter movements of wild waterbirds [6]–[9]. Yet, the effect of physiological changes associated with migration on virus-host interactions, which may interfere with the ability of wild birds to disperse the virus over long distances, is not well understood [8], [10], [11].

Important physiological changes prepare migratory birds for long-distance flights, and are essential for the development and maintenance of the migratory state [12]–[14]. Prior to departure, some migratory bird species increase their body mass by up to 50% of their lean mass [15]. They put on fat stores that will serve as fuel during migratory flights [14]. The relative size of internal organs shift from those accommodating high food consumption rates prior to take–off to those accommodating the actual energy-demanding endurance flights [16], [17]. Behavioral changes are often observed, including migratory restlessness, during which migratory birds display high activity levels [13], [18]. The glucocorticoid stress hormone corticosterone stimulates appetite and locomotion and contributes to regulating these physiological changes in migratory birds [13], [19]. Elevated plasma concentration of corticosterone has been reported in wild songbirds and shorebirds caught just prior to or during migration [12], [13], [18], [20].

When elevated for sustained periods of time, corticosterone is also a potent immuno-suppressor [21]. For example, house sparrows (*Passer domesticus*) treated with corticosterone reactivated latent infection with *Plasmodium relictum*, causing avian malaria [22], [23].Spring relapse of another avian blood parasite, *Haemoproteus danilewskyi*, was associated with high plasma concentration of corticosterone in wild-caught blue jays (*Cyanocitta cristata*) [24]. Similarly, latent infection with the spirochete *Borrelia burgdorferi*, causative agent of Lyme disease, was reactivated upon experimental inducement of the migratory state in redwing thrushes (*Turdus iliacus*) [25]. A higher risk of acquisition or reactivation of infection may have important consequences for the spread of infectious pathogens over long distances. On the one hand, increased probability of acquisition or reactivation of infection may favor the spread of pathogens by migratory birds. On the other hand, increased probability of development of clinical disease may hamper such spread, as infected birds may become unable to undertake migration, or fly long distances [11]. Although stonechats (*Saxicola torquata*) exhibiting variable migratory behavior (from highly migratory to resident) were found equally susceptible to experimental HPAIV H5N1 infection at the time of migration [26], little is known on the impact of corticosterone on influenza virus infection in wild birds.

The life cycle and associated physiological changes of the red knot (*Calidris canutus islandica*, a migratory shorebird), including variations in plasma concentration of corticosterone, have been extensively studied both in the wild and in captivity [16], [27]–[37]. Knots kept in captivity maintain natural body mass cycles [29], [34], even if they are unable to fly beyond the boundaries of the aviary, and show elevated plasma concentration of corticosterone at the time of peak body mass during the spring migration period [13]. Their plasma concentration of corticosterone reaches as high as three times the concentration at the beginning of the body mass increase. They also display migratory restlessness, exhibiting the urge to migrate. These changes reverse at the onset of voluntary fasting, resulting in body mass loss and preceding body and wing molts [38]. Red knots infected with HPAIV H5N1 have not been reported in the wild [39], and these shorebirds are unlikely to be actual spreaders of HPAIV H5N1. However, because of the predictability of the physiological changes associated with the migratory state in captivity in these shorebirds, we used the red knot as a model to assess the effect of elevated plasma concentration of corticosterone associated with the migratory state on infection with HPAIV H5N1.

## Results

### Red knot as a model for infection under the migratory state

In order to demonstrate any effect of corticosterone on infection with HPAIV H5N1 under the migratory state, several prerequisites must be met by the chosen model. First, the bird species must undergo physiological changes associated with migration in captivity, including an increase in plasma concentration of corticosterone. This prerequisite was met by the knot model and results are further detailed below. Second, the bird species must be susceptible to infection with HPAIV H5N1 under non-migratory state. However, it must demonstrate an intermediate susceptibility to the development of clinical disease upon infection, with some birds developing clinical disease and others remaining sub-clinically infected. Such outcome not only depends on the bird species, but also on the infection dose and route of inoculation. This prerequisite is important to maximize our ability to detect any effect of corticosterone on HPAIV H5N1 infection under the migratory state. If all birds develop clinical disease under non-migratory state, an increase in the probability of development of disease may be difficult to detect under the migratory state, since all birds will also likely develop clinical disease. On the other hand, if no bird develops clinical disease under non-migratory state, an increase in the probability of development of disease may not be detected under the migratory state. For example, the infection dose may not be sufficient to result in clinical disease under non-migratory or migratory state alike. This prerequisite was met by the knot model when inoculated intra-esophageally and intra-tracheally with 10^6^ median tissue culture infectious dose (TCID_50_) of HPAIV H5N1. Six knots inoculated in this way during the pre-migration period demonstrated that the bird species, the infection dose and the route of inoculation were appropriate for the aim of the present study. All but one bird in this group became productively infected, and two birds developed clinical disease. Results are further detailed below.

### Migratory state

A total of 29 knots were used in the present study. Experimental infections with HPAIV H5N1 were performed during i) the pre-migration period, in March 2007 (N = 6), ii) the fueling period, in late May 2007 (N = 5), iii) the migration period (when birds are ready to depart) in late May 2009 (N = 9), and iv) the post-migration period, in August 2007, at the time of body and wing molt (N = 6). An additional group of 3 knots were used as negative controls and sham-inoculated during the migration period in 2009. All measures associated with the migratory state, except activity levels and length and mass of organs, were recorded before inoculation. Activity levels were recorded during the inoculation experiments. Length and mass of organs were recorded at necropsy at the end of the experiments. Negative control birds are included in the migration period group for the migratory state analyses.

**Figure 1:**
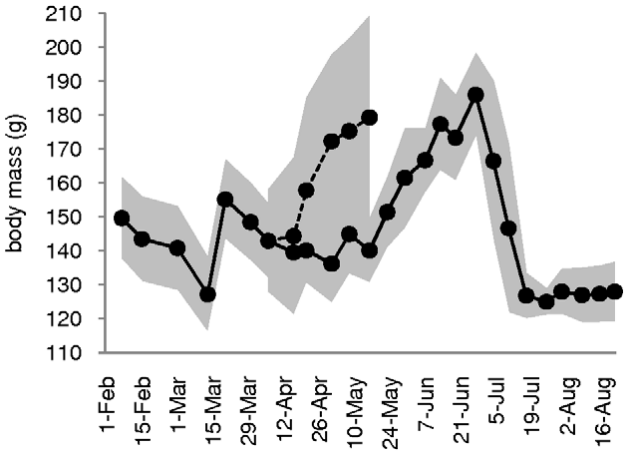
Changes in red knots' pre-inoculation body mass between February and August. Plain line: pre-inoculation body mass of knots inoculated in 2007; dashed line: pre-inoculation body mass of knots inoculated in 2009; grey shade: standard deviation. The number of knots inoculated in 2007 decreases with time as birds are used in experiments (N = 17 from 1 February to 16 March; N = 11 from 17 March to 30 May; N = 6 from 31 May to 22 August). The number of knots inoculated in 2009 includes 9 knots and 3 negative control knots from 7 April to 28 May.

Overall, the birds underwent the physiological changes associated with migration. They maintained their natural (pre-inoculation) body mass cycle between February and August (Fig. 1). However, the peak in body mass associated with the migratory state occurred approximately one month later than expected in 2007. Therefore, the group of 5 knots inoculated in late May 2007 had not reached the peak in body mass, and was in the early stage of body mass gain associated with early migratory state (fueling period). At the time of infection, the pre-inoculation body mass of these knots was higher than that of the knots inoculated during the pre- and post-migration periods (Table 1). An extra group of 12 knots in migratory state was added in May 2009. The pre-inoculation body mass of these 12 knots inoculated or sham-inoculated at the time of migration was reaching a plateau in late May 2009, as expected (migration period; Fig. 1). At the time of inoculation, their body mass was higher than that of the knots inoculated during the pre- and post-migration periods (Table 1).

**Table 1:** Significant differences in migratory state and constitutive immunity in red knots inoculated with highly pathogenic avian influenza virus (HPAIV) H5N1.

	Level of significance per comparison between periods		
Parameter	Fueling period	Migration period	Post-migration period
Pre-inoculation body mass	II>I, p = 0.03	III>I, p = 0.003	
	II>IV, p = 0.03	III>IV, p = 0.004	
Pre-inoculation plasma concentration of corticosterone		III>II, p = 0.03	
		III>IV, p = 0.001	
Relative mass of gizzard	II<I, p = 0.004		
Relative mass of liver	II<IV, p = 0.008	III<IV, p = 0.001	
Relative mass of intestinal tract	II<I, p = 0.004		
	II<IV, p = 0.008		
Relative length of intestinal tract	II<I, p = 0.004		
Activity levels	II>I, p = 0.009	III>I, p<0.0001	
		III>IV, p = 0.001	
White blood cell count	II<IV, p = 0.01	III<IV, p = 0.03	
Lymphocyte count	II<IV, p = 0.008	III<IV, p = 0.006	
Lymphocyte proportion	II<I, p = 0.009	III<I, p = 0.001	
		III<IV, p = 0.009	
*Escherichia coli* killing			IV<I, p = 0.02
			IV<III, p = 0.01
Hemolysis			IV>I, p = 0.004
			IV>II, p = 0.01
			IV>III, p = 0.006

I: knots inoculated during the pre-migration period (N = 6); II: knots inoculated during the fueling period (N = 5); III: knots inoculated during the migration period (N≤12); IV: knots inoculated during the post-migration period (N = 6). The three negative control knots are included in the migration period group (III) for the analyses of pre-inoculation measures of body mass, constitutive immunity and plasma concentration of corticosterone, and of the measures of activity levels (N = 12); the necropsied negative control knot is included in the migration group (III) for the analyses of the measures of length and mass of organs (N = 10).

**Figure 2:**
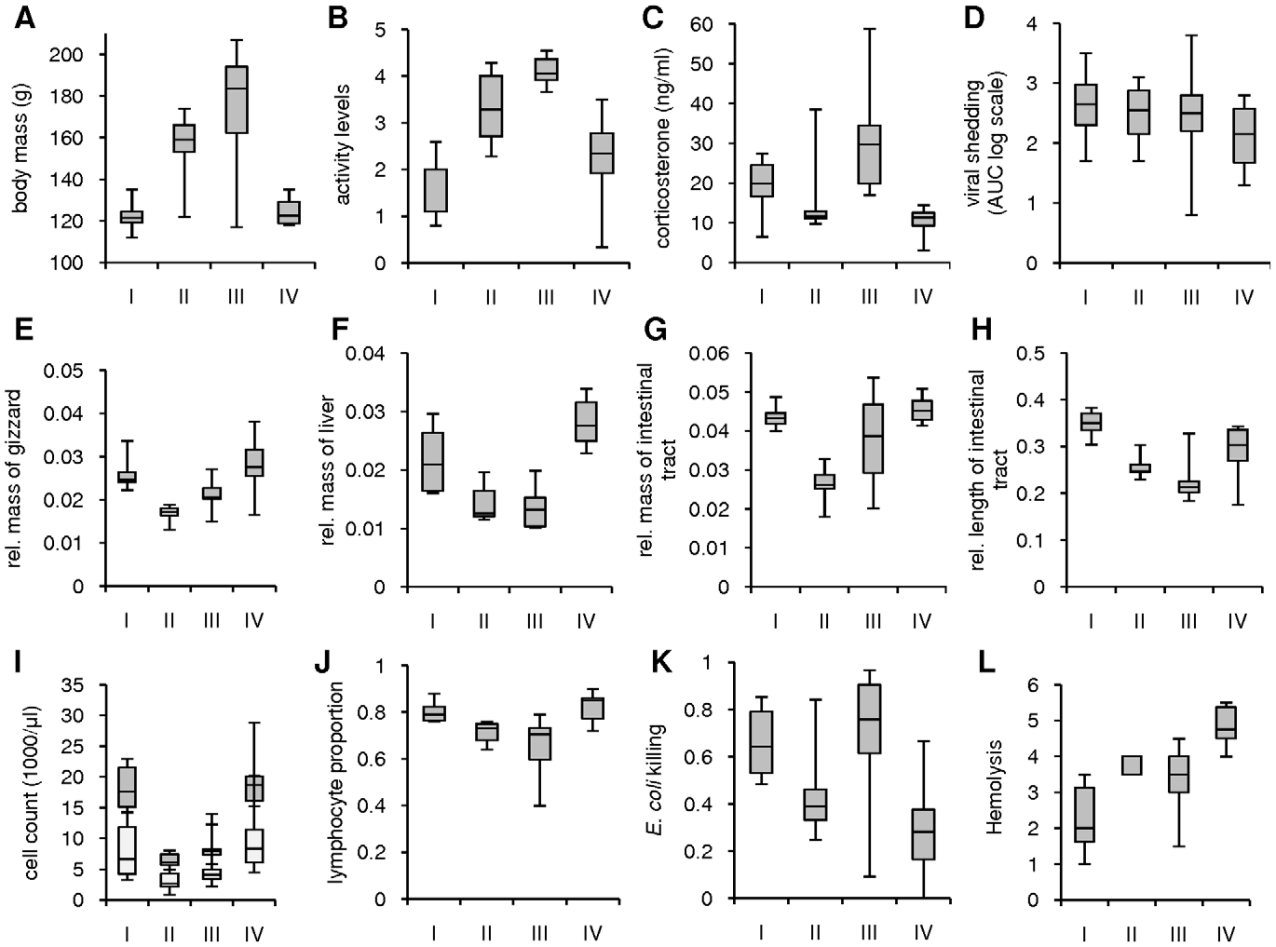
Migratory state, constitutive immunity and viral shedding of highly pathogenic avian influenza virus (HPAIV) H5N1. Knots were inoculated during the pre-migration period (I; N = 6), the fueling period (II; N = 5), the migration period (III; N≤12), and the post-migration period (IV; N = 6). Results are shown for pre-inoculation body mass (A), activity levels (B), pre-inoculation plasma concentration of corticosterone (C), viral shedding of HPAIV H5N1 from the pharynx (AUC: area under the curve; D), relative mass of the gizzard (E), liver (F), and intestinal tract (G), relative length of the intestinal tract (H), pre-inoculation white blood cell count (dark grey) and lymphocyte count (light grey; I), pre-inoculation proportion of lymphocytes (J), pre-inoculation *Escherichia coli* killing (K) and pre-inoculation hemolysis (L). rel. = relative. Box lower and higher limits are first and third quartiles, middle line is the median, and lower and upper whiskers are minimum and maximum values. Negative control knots are included in the analyses of pre-inoculation measures of body mass, constitutive immunity and plasma concentration of corticosterone, and of the measures of activity levels (N = 12), and the necropsied negative control knot is included in the analyses of the length and mass of organs (N = 10). For viral shedding, non-productively infected knots are not included (I: N = 5; II: N = 4; III: N = 5; IV: N = 4). Significant differences and p values are given in Table 1.

After correcting for body mass, red knots inoculated during the fueling and migration periods had generally lighter or shorter organs associated with the digestive tract, including gizzard, intestinal tract and liver, than the knots inoculated during the pre- and post-migration periods (Fig. 2E–H).Significant differences and p values are given in Table 1. The mass of the pectoral muscles, heart and brain corrected for body mass did not differ between the four groups of knots.

Behaviour was recorded during the inoculation experiments every 3 min during 15 min every day, and categorized into active or inactive behaviors. Daily ratios of active behaviors were calculated and averaged for each knot over the duration of the experiments. There was no significant difference in the activity levels of the knots inoculated with HPAIV H5N1 during the migration period and those of the three negative control knots, thus the negative control knots were included in the migration period group. Red knots displayed active behaviors more often during the fueling period and during the migration period than during the pre- and post-migration periods (Fig. 2B; Table 1).

Pre-inoculation plasma concentration of corticosterone did not differ between the three groups of knots inoculated in 2007, with an overall mean of 14.6 ng/ml (SD = 7.7). It was significantly higher in knots inoculated during the migration period in 2009, reaching a mean of 34.5 ng/ml (SD = 15, p = 0.001, Fig. 2C). These values were well within the range of those measured in free-living red knots [19]. There was a positive correlation between pre-inoculation body mass and plasma concentration of corticosterone (rho = 0.28, p = 0.02), and a negative correlation between the relative mass of the knots' liver and pre-inoculation plasma concentration of corticosterone (rho = −0.41, p = 0.02).

### Constitutive immunity

Measures of constitutive immunity included pre-inoculation white blood cell counts, bacteria killing capacity of whole blood against *Escherichia coli* and *Staphylococcus aureus*, and hemolysis-hemagglutination properties of knot plasma. The pre-inoculation white blood cell counts, proportion and concentration of lymphocytes, *E. coli* killing, and hemolysis differed between the four groups of knots (Fig. 2I–L). The variations in pre-inoculation white blood cell counts reflected variations in the number of lymphocytes, as these made on average between 70% and 82% of the white blood cell counts. The pre-inoculation proportion and concentration of lymphocytes were lower in knots inoculated during the fueling period and during the migration period than in knots inoculated during the pre- and post-migration periods (Table 1). Pre-inoculation *E. coli* killing was weakest in knots inoculated during the post-migration period, while pre-inoculation hemolysis mediated by the complement and other soluble proteins was strongest in these birds. Hemolysis positively correlated with the relative mass of the liver (rho = 0.41, p = 0.01). None of the constitutive immunity parameters correlated with pre-inoculation plasma concentration of corticosterone.

### Clinical signs of infection

Five knots developed clinical disease or died during the experiment. Two knots inoculated during the pre-migration period exhibited severe disease, including apathy and neurological signs, such as loss of balance and tremors, and were euthanized at 5 and 6 days post inoculation (dpi). One knot inoculated during the fueling period presented torticollis and was euthanized at 6 dpi. One knot inoculated during the migration period was found dead at 11 dpi without showing clinical signs beforehand. One knot inoculated during the post-migration period exhibited neurological signs and died at 2 dpi. It had a co-infection with HPAIV H5N1 and coccal bacteria and was further excluded from the analysis because the bacterial infection contributed to severe disease and rapid death of the bird. The appearance of clinical signs in these knots was sudden and the affected birds did not behave significantly differently on the preceding days than birds that remained sub-clinically infected.

Body mass loss was observed in red knots inoculated with HPAIV H5N1 and negative control birds. Overall, there was no correlation between body mass loss and the level of viral shedding. Likewise, body mass loss did not differ between knots with clinical disease and sub-clinically infected knots; or between knots productively infected and knots that remained uninfected or negative control knots. Body mass loss was positively correlated with body mass at the time of inoculation or sham-inoculation (rho = 0.68, p<0.0001), irrespective of the infection status of the birds.

### Viral shedding

**Figure 3:**
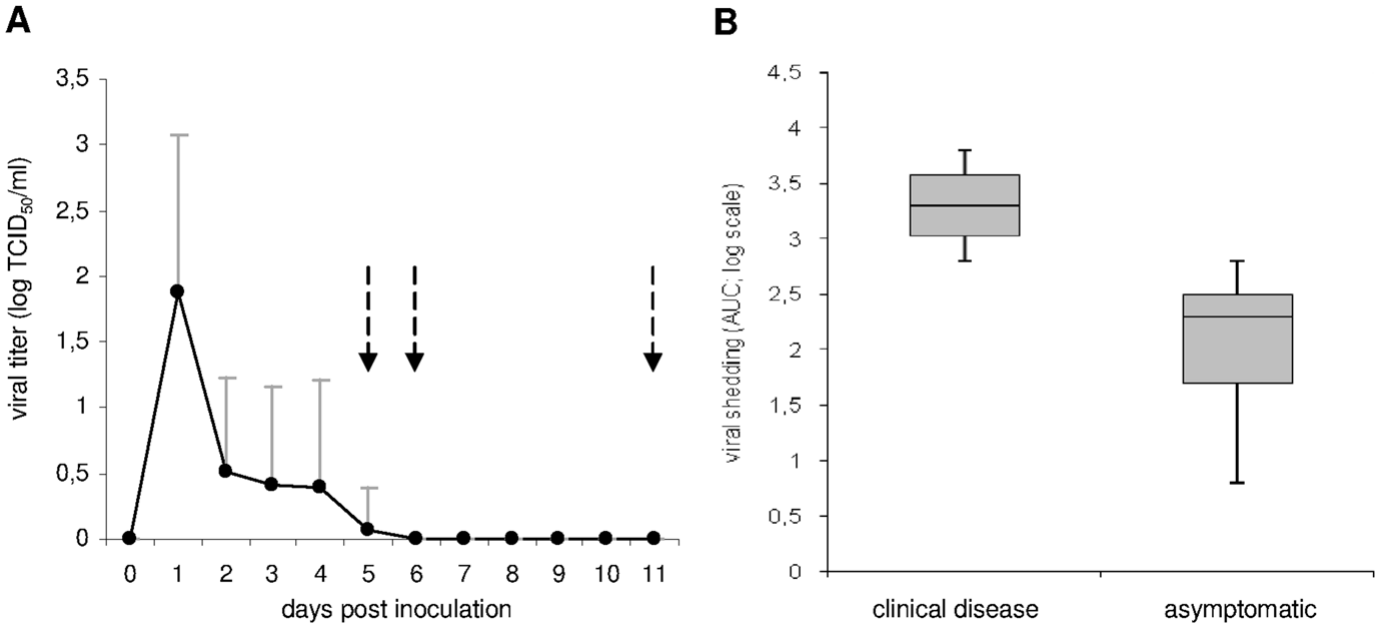
Clinical disease and viral shedding of highly pathogenic avian influenza virus (HPAIV) H5N1 from the pharynx of infected knots. Mean viral titers of productively infected knots (N = 17) are shown (A); days of onset of clinical disease are marked by arrows. Bars represent the upper standard deviation. Red knots presenting clinical disease (N = 4) shed higher titers of HPAIV H5N1 than productively infected knots that remained asymptomatic (B; N = 13; p = 0.005). Box lower and higher limits are first and third quartiles, middle line is the median, and lower and upper whiskers are minimum and maximum values. AUC: area under the curve.

Red knots infected with HPAIV H5N1 shed virus from the pharynx during 1 to 5 dpi, with peak viral titers of up to 10^3.8^ TCID_50_/ml at 1 to 3 dpi (Fig. 3E). HPAIV H5N1 RNA was detected in pharyngeal swabs up to 6 dpi as measured by real-time reverse-transcription polymerase chain reaction (RT-PCR). The virus was not isolated from any cloacal swabs, although HPAIV H5N1 RNA was occasionally detected in cloacal swabs up to 6 dpi. No virus was isolated from any swabs of the negative control knots. The level of viral shedding was determined for each bird by calculating the area under the viral shedding curve. There was no statistical difference in the level or duration of viral shedding between the four groups of knots (Fig. 2D). The knots that presented severe neurological disease or sudden death following inoculation shed higher pharyngeal viral titers than the birds that remained sub-clinically infected (p = 0.005; Fig. 3F). No other statistical difference between sick and sub-clinically infected birds was detected.

One knot inoculated during the pre-migration period, one knot inoculated during the fueling period, four knots inoculated during the migration period, and one knot inoculated during the post-migration period did not shed HPAIV H5N1. The proportion of non-productively infected knots did not differ between groups (Freeman-Halton extension of the Fisher's exact test, p = 0.8, [40]).During the migration period, the four knots that did not shed virus had lower plasma concentrations of corticosterone than the birds that did shed virus in this group (p = 0.05).

**Figure 4:**
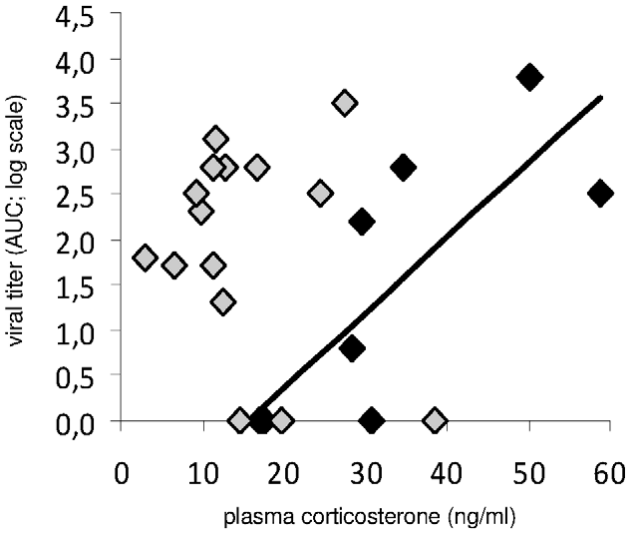
Viral shedding of highly pathogenic avian influenza virus (HPAIV) H5N1 and plasma concentration of corticosterone. The level of viral shedding from the pharynx and plasma concentration of corticosterone in red knots are positively correlated only in knots inoculated during the migration period (black diamonds; rho = 0.80, p = 0.004). No correlation was detected in knots infected outside the migration period (grey diamonds), or overall for all birds taken together. AUC: area under the curve.

Overall, when all birds were considered together, the level of viral shedding from the pharynx did not correlate with pre-inoculation plasma concentration of corticosterone. However, at within-group level, the level of viral shedding from the pharynx was positively correlated with pre-inoculation plasma concentration of corticosterone in knots infected during the migration period (rho = 0.80, p = 0.004; Fig. 4). The level of viral shedding from the pharynx did not correlate with any other physiological or constitutive immunity data.

### Viral isolation from internal organs

HPAIV H5N1 was isolated from the trachea (10^3.2^ TCID_50_/g), lungs (10^1.5^ TCID_50_/g), air sac (10^3.5^ TCID_50_/g), duodenum (10^0.8^ TCID_50_/g), pancreas (10^5.2^ TCID_50_/g), heart (10^0.8^ TCID_50_/g) and kidney (10^2.5^ TCID_50_/g) of the knot with clinical disease inoculated during the pre-migration period and that was euthanized at 5 dpi. No virus was isolated from any organs of the other clinically affected knot from that group. The virus was isolated from the brain (10^5.2^ TCID_50_/g) of the knot inoculated during the fueling period and that presented torticollis at 6 dpi. The virus was isolated from the brain (10^3.8^ TCID_50_/g) of the knot inoculated during the migration period and that died at 11 dpi. No virus was isolated from any organs of the knots showing no clinical disease, which were euthanized at 11 dpi, or from any organs of the negative control bird.

### Gross pathology

One knot inoculated during the fueling period and that showed clinical signs had mild diffuse acute pancreatic congestion. All other knots inoculated with HPAIV H5N1, and the negative control knot had no significant gross lesions.

### Histopathology

**Figure 5:**
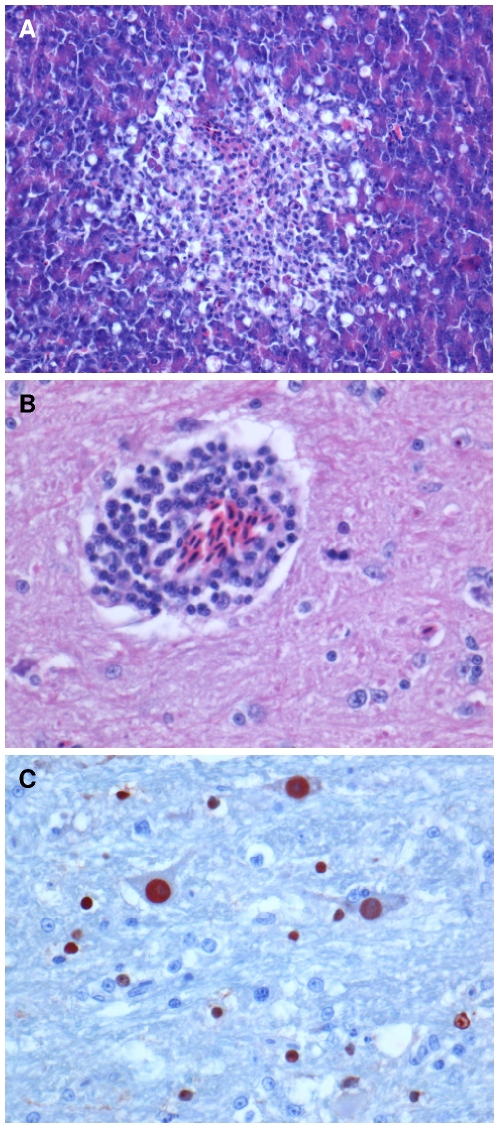
Pathology of highly pathogenic avian influenza virus H5N1 infection in red knots. Lesions of acute pancreatitis, showing a discrete focus of pancreatic exocrine cell necrosis (A); lesions of acute encephalitis, showing perivascular accumulation of lymphocytes (B); and presence of influenza virus antigen in nuclei of neurons (large cells) and glial cells (small cells; C). Slides were stained with hematoxylin and eosin (A and B), or by immunohistochemistry using a monoclonal antibody against the nucleoprotein of influenza A virus as the primary antibody (C).

The four knots that presented clinical disease or died during the experiment had multiple foci of severe acute encephalitis, characterized by neuronal chromatolysis, perivascular cuffing, neuronophagia, neuropil vacuolisation and foci of gliosis in the brainstem, cerebellum and cerebrum (Fig. 5A). Loss of Purkinje cells was observed in the cerebellum. Similar lesions of necrosis and inflammation were seen in the cervical spinal cord of the bird with torticollis. One bird with clinical signs had multiple foci of acute necrotizing pancreatitis, associated with heterophilic infiltrates (Fig. 5B). One knot inoculated during the post-migration period that showed no clinical signs had multiple foci of perivascular cuffing in the cerebrum. No significant lesions were observed in any organ of any other knot.

### Immunohistochemistry

The lesions in the brain of the knots that presented clinical disease or died during the experiment were associated with the presence of influenza virus antigen in neurons, glial cells, ependymal cells, or a combination of these, as determined by red diffuse staining in the nucleus and to a lesser extent the cytoplasm of these cells by immunohistochemistry (Fig. 5C). In one bird with clinical disease, influenza virus antigen was detected in the nuclei of a few pancreatic exocrine cells in the periphery of necrotic lesions in the pancreas. In another bird with clinical disease, influenza virus antigen was detected in mononuclear cells—identified as macrophages—in the liver and spleen. Influenza virus antigen was not detected in any organ of the knots that did not show clinical signs or die, or in any organ of the negative control knot.

## Discussion

The results of this study show that, at the between-group level, red knots inoculated during the pre-migration period, fueling period, migration period and post-migration period appeared equally susceptible to infection with HPAIV H5N1. The levels of viral shedding, the proportion of birds that acquired infection and the proportion that developed clinical disease were similar in each group. Taking all birds together, pre-inoculation plasma concentration of corticosterone did not correlate with probability of acquisition of infection, probability of development of clinical disease, level of viral shedding, or parameters of constitutive immunity. In particular, knots with high levels of corticosterone during the migration period did not shed higher viral titers than knots inoculated during pre-migration, fueling or post-migration periods. These results are similar to those reported by Kalthoff and colleagues where stonechats exhibiting different migratory behavior were shown equally susceptible to experimental infection with HPAIV H5N1 at the time of migration [26]. Therefore, the risk of HPAIV H5N1 infection in wild birds and of potential transmission between wild birds and poultry may be similar at different times of the year, irrespective of wild birds' migratory status.

However, at the within-group level, there was a significant positive correlation between pre-inoculation plasma concentration of corticosterone and the level of viral shedding from the pharynx in knots inoculated during the migration period. Furthermore, the birds within this group that were productively infected (5 out of 9) had significantly higher pre-inoculation plasma concentration of corticosterone than those that were not productively infected. To our knowledge, this is the first report of an association between plasma concentration of corticosterone and severity of viral infection in birds. This finding indicates that high levels of corticosterone during the migration period may be associated with increased probability of acquisition of infection with HPAIV H5N1 and higher levels of viral shedding. Because birds with high levels of corticosterone during the migration period are physiologically ready for take-off and long-distance flight [13], increased probability of acquisition of infection and higher viral shedding in these birds may favor long-distance spread of the virus over large geographical areas.

The mechanism for increased probability of acquisition of infection during the migration period when levels of corticosterone are high is unclear. There may be an association with lymphocyte counts in the blood, which were significantly lower during the migration period: B and T lymphocytes are known to be essential in controlling avian influenza virus infection in birds [41]. However, pre-inoculation lymphocyte counts did not correlate with plasma concentration of corticosterone or viral shedding in this study. It is possible that additional factors associated with the migratory state, and in particular with elevated plasma concentration of corticosterone, may affect the knots' probability of acquiring infection at the time of migration.

Elevated pre-inoculation plasma concentration of corticosterone did not result in an increase in the probability of development of clinical disease. One to two birds developed neurological disease and died of the infection in each group, including during the migration period. Based on the proportion of birds that presented clinical signs under non-migratory state, our experimental design would have allowed to detect a three- to four-fold increase in probability of development of clinical disease during the migration period, with a statistical power of 80% or more, using the statistical power test for 2 proportions. Therefore, birds in migratory state, with elevated plasma concentration of corticosterone, may not be more prone to develop clinical disease following HPAIV H5N1 infection than birds under non-migratory state.

A number of productively infected, non-productively infected, and negative control knots showed body mass loss during the experiment, while others, including productively infected knots that shed high viral titers, maintained or gained body mass. There was no association between body mass loss and infection in the present study. Thus, body mass loss was likely associated and triggered by the experimental procedures (e.g., transport and change in environment) and not by infection with HPAIV H5N1. However, body mass loss associated with HPAIV H5N1 infection may not have been detected due to the small sample size and individual variations in knots' response to infection.

Red knots that developed clinical disease had higher levels of viral shedding from the pharynx than knots that remained sub-clinically infected. This has also been described in other experimentally infected waterbirds [4]. Clinically affected red knots developed severe neurological disease, associated with viral replication in neurons and other cells of the central nervous system, demonstrating neurotropism of HPAIV H5N1 in this species. Neurological disease has been described in wild birds following natural and experimental infection with HPAIV H5N1, in association with viral replication in brain neurons [2]–[4], [42], [43]. Importantly, affected knots suddenly developed clinical disease at 5 to 11 dpi, and did not show visible changes in behavior on the preceding days. It is unlikely that even minor clinical signs in these knots would have been missed during the daily behavioral observations of 15 min duration, because of their high level of activity and the nature of their behavior (e.g., sand probing). Likewise, most other species of experimentally infected birds that developed clinical disease upon infection with HPAIV H5N1 showed debilitating signs of disease at 4 to 8 dpi, without visible signs during the preceding days [2]–[4]. Shedding of HPAIV H5N1 by experimentally infected knots lasted from 1 to 5 dpi and peaked at 1 to 3 dpi. This is comparable to other species of experimentally infected birds, which typically shed virus up to 4 or 5 dpi with a peak at 1 to 2 dpi [2]–[4]. Therefore, a major proportion of viral shedding occurs while birds are clinically normal and apparently able to fly, thus potentially allowing for the long-distance dispersal of HPAIV H5N1. Nevertheless, any insults to the health of migratory birds that are ready for potentially dangerous and extremely demanding long-distance migration [17] may prevent them from undertaking migration at all [44]. Thus, it remains to be shown in free-living migratory birds whether sub-clinical infection reduces the likelihood of taking off for long migratory journeys. Another caveat that cannot be addressed by the present study is the fact that knots under experimental settings were fed *ad libitum*, which may differ from conditions met in nature.

In conclusion, the risk of HPAIV H5N1 infection in wild birds and of potential transmission between wild birds and poultry may be similar at different times of the year, irrespective of wild birds' migratory status. However, during the migration period, migratory birds may be more susceptible to acquisition of infection with HPAIV H5N1 and shed higher viral titers when ready to undertake migration (i.e., when displaying elevated plasma concentration of corticosterone), but not be more susceptible to the development of clinical disease, which starts at the end or after the period of viral shedding. Assuming that sub-clinical infection does not affect the likelihood of migratory take-off, this may favor the geographical spread of the virus by migratory birds during the migration period, and increase the chance of virus incursion into wild or domestic bird populations along migratory pathways, resulting in epidemics. This possibly occurred during the HPAIV H5N1 outbreaks in Europe, the Middle East and Africa in winter 2005–2006 [6]–[9]. Similarly, since fall 2010, HPAIV H5N1 outbreaks have been reported in wild birds in Japan, preceding recurring outbreaks in poultry [45]. The implications of these results are that prevention of contact between poultry and wild birds is essential all-year round to prevent cross-species transmission of HPAIV H5N1 between wild birds and poultry, and is especially important at the time of migration in order to reduce long-distance spread of the virus to novel geographic areas.

## Materials and Methods

### Ethics statement

This study was carried out in strict accordance with European guidelines (EU directive on animal testing 86/609/EEC) and Dutch legislation (Experiments on Animals Act, 1997). The protocol was approved by the Committee on the Ethics of Animal Experiments of the Dutch Royal Academy of Science (DEC of KNAW; Permit Number: NIOZ 04.03), and by the Committee on the Ethics of Animal Experiments of the Erasmus Medical Centre (DEC of EMC; Permit Numbers: EMCNr. 1068 122-07-01 and EMCNr. 1745 122-09-06). Animal welfare was observed on a daily basis, and all experimental procedures were performed under anesthesia with isoflurane to minimize animal suffering.

### Red knots

Twenty-nine 2-to-3-year-old red knots, captured with mist nets near high tide roosts in the Dutch Wadden Sea and along the French Atlantic coast in late July 2006 and October 2008, were kept in aviaries at the Royal Netherlands Institute for Sea Research (NIOZ) as described [13], [20] until the start of the inoculation experiments in 2007 (N = 17) and 2009 (N = 12), respectively. There, they were exposed to natural seasonal photoperiods. Once per week, the birds' body mass was measured and the extent of molt was scored [13], [20]. The birds were shipped to the Department of Virology of the Erasmus Medical Centre one day before the start of the experimental procedures. Care was taken to subject all knots from both cohorts to the same capture, handling, housing, transport and experimental conditions. In particular, all birds were held in captivity at the NIOZ aviaries under the same conditions for 8 to 12 months after capture with mist nests at the end of summer/beginning of fall, and before the start of the experiments; they were inoculated with virus originating from the same batch.

### Highly pathogenic avian influenza virus H5N1

A virus stock of influenza virus A/turkey/Turkey/1/2005 (H5N1) was prepared by two passages in 10-day-old embryonated chicken eggs. The harvested allantoic fluid was titrated on Madin-Darby canine kidney (MDCK) cells according to standard methods [46] and reached an infectious virus titer of 10^8.4^ TCID_50_/ml. It was diluted with phosphate-buffered saline (PBS) to obtain a final titer of 10^6.5^ TCID_50_/ml. All experiments with HPAIV H5N1 were performed under Biosafety Level 3+ conditions.

### Experimental design

Experimental infections of red knots with HPAIV H5N1 were performed during i) the pre-migration period, in March 2007 (N = 6), ii) the fueling period, in late May 2007 (N = 5), iii) the migration period in late May 2009 (N = 9), and iv) the post-migration period, in August 2007, at the time of body and wing molt (N = 6). An additional group of 3 knots were used as negative controls and sham-inoculated during the migration period in 2009. The group inoculated in late May 2007 was retrospectively shown to have not reached the peak in pre-inoculation body mass and plasma concentration of corticosterone (hence qualified as fueling period); the group inoculated in late May 2009 did reach the plateau in pre-inoculation body mass and plasma concentration of corticosterone (hence qualified as migration period; see results above).

Between 5 and 7 days before infection, the knots were bled from the brachial vein into heparinized capillary tubes, to collect about 600 µl of blood. Blood smears were done immediately after sampling and the remaining blood was further processed within an hour of sampling, for the measurement of constitutive immunity (see below). The assays of immune function used in this study are robust to periods of capture and handling stress of at least 30 min [47] and all samples were taken within 20 min of entering the aviaries.

Between 3 and 5 days before infection, the knots were bled from the brachial vein into heparinized capillary tubes, to collect 200 to 300 µl of blood. Blood was sampled and centrifuged, and plasma was stored at −80°C until the samples were further processed for the measurement of plasma concentration of corticosterone (see below). Plasma concentration of corticosterone rises in samples collected 3 min or more after entry of people into the aviary, due to capture and handling stress, thus only samples taken within 3 min of entry into the aviary were used to obtain baseline values of plasma concentration of corticosterone [13], [48].

One day before infection, the knots were shipped to the Erasmus Medical Centre. There, serum samples were collected and analyzed by using a competitive enzyme-linked immune-sorbent assay (ELISA) to determine whether the birds had been previously infected with influenza A virus. Serum was analyzed by use of a commercially available influenza A antibody ELISA kit for the detection of antibodies against the nucleoprotein of influenza A virus (European Veterinary Laboratory, Woerden, The Netherlands) according to the manufacturer's instructions. The sensitivity and specificity of this test are unknown for red knots. One red knot inoculated during the pre-migration period and two knots inoculated during the migration period had prior antibodies against influenza A virus. The knot inoculated during the pre-migration period that had prior antibodies against influenza virus did not become productively infected. In contrast, the two knots inoculated during the migration period that had prior antibodies against influenza virus became productively infected and shed virus titers similar to those of the birds of that group without prior antibodies (p = 0.7). Because the presence of prior antibodies against influenza virus was not associated with detectable protection against HPAIV H5N1 infection and did not impact on the levels of viral shedding, we included these birds in the study.

Groups of three to six knots were placed in one negatively-pressurized isolator unit, adapted to house these shorebirds. The floor of the unit was made of a fine gridded mesh, adapted to the size of the birds' feet. A shallow basin filled with sand and water was added to satisfy the knots' probing behavior. Another shallow basin filled with water was added for bathing. The knots were fed *ad libitum* with protein-rich trout food pellets [13] and a water fountain was provided. Lighting was kept on daily for 12 hours during the pre-migration period, 17 hours during the fueling and migration periods, and 14 hours during the post-migration period, to reproduce the seasonal photoperiods.

On day 0, the knots were inoculated intra-esophageally and intra-tracheally by use of a catheter with 10^6^ TCID_50_ of HPAIV H5N1 in a volume of 0.3 ml via each route. The 3 negative control birds were sham-inoculated in the same manner with PBS, and housed in a separate isolator. All birds were observed daily for clinical signs, such as apathy, respiratory, intestinal and neurological signs. In addition, the behavior of each knot was recorded every 3 min during 15 min every day, when the birds were undisturbed and unaware of the presence of the investigator. The body mass was measured and pharyngeal and cloacal swabs were collected daily from day 0 to day 8 and every 2 days thereafter. Pharyngeal and cloacal swabs were placed in 3 ml of virus transport medium (Hank's balanced salt solution containing 10% glycerol, 200 U/ml penicillin, 200 µg/ml streptomycin, 100 U/ml polymyxin B sulphate, 250 µg/ml gentamycin), and kept on ice. After vigorously shaking the tubes containing the swabs with a test tube shaker, 200 µl of the suspension were sampled and added to 300 µl of lysis buffer for RNA isolation and RT-PCR. The remainder was frozen at −80°C until viral titration. Knots inoculated with HPAIV H5N1 were euthanized by exsanguination under anesthesia with isoflurane, when pharyngeal and cloacal swabs were determined negative by RT-PCR on two consecutive sampling days, or when severe clinical signs were observed. One of the three negative control birds was euthanized by exsanguination under anesthesia with isoflurane on the same day as the birds inoculated during the migration period, while the remaining two were shipped back to NIOZ at the end of the experiment.

Six 4-to-6-week-old white leghorn chickens were inoculated intra-esophageally and intra-tracheally by use of a catheter with 10^6^ TCID_50_ of HPAIV H5N1 in a volume of 0.3 ml via each route. They served as positive controls to confirm the pathogenicity of the HPAIV H5N1 batch. They were euthanized by exsanguination under anesthesia with isoflurane two days following infection or when severe clinical signs were observed. Inoculation resulted in systemic HPAIV H5N1 infection in chickens (data not shown), that showed severe clinical signs within 48 hours of inoculation. This demonstrated that the batch had retained full pathogenicity.

### Measures of pre-inoculation constitutive immunity

Constitutive immunity was measured as described by Buehler and colleagues [28]. Briefly, the bacteria killing capacity of whole blood against *E. coli* and *S. aureus* was assessed by incubating diluted blood samples with *E. coli* suspension for 10 min, or with *S. aureus* suspension for 120 min at 41°C, and inoculating the mixtures onto agar plates in duplicate. Control plates were inoculated with *E. coli* or *S. aureus* suspension. The plates were incubated at 36°C overnight and the colonies were counted the next day. The proportion of killed microorganisms was calculated as the number of colonies on the test plates relative to the number of colonies on the control plates.

White blood cell counts were performed on the knots' blood smears examined by light microscope at 1000× magnification with oil immersion. The first 100 white blood cells were counted and classified as heterophils, eosinophils, lymphocytes, or monocytes. In addition, the number of thrombocytes seen during the white blood cell counts was recorded as an estimate of the relative number of thrombocytes per white blood cell. In combination with the blood smears, white blood cell concentrations were obtained using the indirect eosinophil Unopette method [49].

The hemolysis-hemagglutination assay was performed as described by Matson and colleagues [50]. Briefly, the hemolytic and hemagglutinating abilities of the knots' plasma were assessed by incubating serial dilutions of the plasma samples with 1% suspension of rabbit red blood cells in 96-well plates, at 41°C for 90 min. The plates were scanned for hemagglutination (mediated by pre-existing antibodies) after 20 min, and for hemolysis (mediated by the complement and other lytic plasma proteins) after 90 min.

### Measure of pre-inoculation plasma concentration of corticosterone

Total plasma concentration of corticosterone was determined using an enzyme-immunoassay [51], [52] following Müller and colleagues [53]. Corticosterone in 10 µl plasma and 190 µl water was extracted with 4 ml dichlormethane, re-dissolved in PBS and given in triplicates in the enzyme-immunoassay. The dilution of the corticosterone antibody (Chemicon; cross-reactivity: 11-dehydrocorticosterone 0.35%, progesterone 0.004%, 18-OH-DOC 0.01%, cortisol 0.12%, 18-OH-B 0.02% and aldosterone 0.06%) was 1∶8000. We used horseradish peroxidase (1∶400000) linked to the corticosterone antibody as enzyme label and 2,2′-Azino-*bis*(3-ethylbenzo-thiazoline-6-sulfonicacid)diammonium salt as substrate. The analyses were done in one assay on three plates. The concentration of corticosterone in plasma samples was calculated by using the standard curve run in duplicate on each plate. Plasma from chickens with two different corticosterone concentrations was included as internal control on each plate in duplicate. The coefficient of variation was 13.4% for the lower control, and 5.0% for the upper control.

### Pathology and immunohistochemistry

Necropsies and tissue sampling were performed on all inoculated knots, one negative control knot, and three inoculated chickens according to a standard protocol. Internal organs were examined for lesions and sampled. The mass and length of the knots' emptied intestinal tract (from duodenum to cloaca), and the mass of the knots' pectoral muscle, emptied gizzard, liver, heart, and brain were measured. The mass of the organs was corrected for body mass by dividing the mass values of each organ by the bird body mass. After fixation in 10% neutral-buffered formalin and embedding in paraffin, tissue sections were stained with hematoxylin and eosin for histological evaluation or with an immunohistological method using a monoclonal antibody against the nucleoprotein of influenza A virus as a primary antibody for detection of influenza viral antigen [54]. Lung tissue of a chicken experimentally infected with influenza virus A/turkey/Turkey/1/2005 (H5N1) was included as a positive control. Isotype-matched and omission controls were included as negative controls. The following tissues were examined by these two methods: trachea (3 sections), lungs (3 sections), air sac, duodenum and pancreas (3 sections), jejunum (3 sections), ileum and cecum (3 sections), colon (3 sections), spleen, heart, liver, kidney, adrenal gland, and brain (2 sections).

### RNA isolation and RT-PCR

RNA isolation and RT-PCR were performed on the swab suspensions stored in lysis buffer as described [55]. Briefly, RNA was isolated by using a MagnaPure LC system with the MagnaPure LC Total Nucleic Acid Isolation Kit (Roche Diagnostics, Almere, the Netherlands). Real-time RT-PCR assays were performed on an ABI Prism 7000 Sequence Detection System machine (Applied Biosystems, Foster City, CA, USA) by using the TaqMan EZ RT-PCR Core Reagents Kit (Applied Biosystems, Nieuwerkerk a/d IJssel, the Netherlands) according to the manufacturer's instructions. The test used a hybridization probe (5′-6-FAM-TTT-GTG-TTC-ACG-CTC-ACC-GTG-CC-TAMRA-3′) and specific primers (forward: 5′-AAG-ACC-AAT-CCT-GTC-ACC-TCT-GA-3′ and reverse: 5′-CAA-AGC-GTC-TAC-GCT-GCA-GTC-C-3′) to detect the matrix gene of HPAIV H5N1. For each run, the samples were prepared and processed in parallel with two negative and positive control samples. A Ct value of 40 or more was considered negative.

### Viral isolation

The same tissues examined for histopathology were sampled for viral titration. Tissue samples were weighed and homogenized in 3 ml of transport medium with a homogenizer (Kinematica Polytron, Lucerne, Switzerland). Ten-fold serial dilutions of all tissue homogenates and of the swab suspensions that were determined positive by RT-PCR were inoculated into MDCK cells in triplicate as described previously [46]. The minimal detectable titer was 10^0.8^ TCID_50_/ml.

### Statistical analysis

Statistical differences in the sizes of internal organs, viral shedding titers, constitutive immunity data or plasma concentration of corticosterone between the four groups of inoculated knots were assessed with the non-parametric Kruskal-Wallis test. In case of significant differences as detected by the Kruskal-Wallis test, statistical differences between two groups were assessed with the non-parametric Mann-Whitney test. Correlations between the sizes of internal organs, viral shedding titers, constitutive immune data and plasma concentration of corticosterone were assessed with the non-parametric Spearman rank correlation test. Bonferonni correction was used when multiple statistical comparisons were done on one set of data. Differences were considered significant when p≤0.05 before Bonferonni correction.

The present sample design would allow to detect a three- to four-fold increase in probability of acquisition of infection or of development of clinical disease during the migration period compared to birds under non-migratory state, with a statistical power of 80% or more, using the statistical power test for 2 proportions. It is possible that subtler differences were not detected due to small group sizes.
